# High-level of anxiety and depressive symptoms among patients with general medical conditions and community residents: a comparative study

**DOI:** 10.1186/s12888-021-03336-6

**Published:** 2021-06-30

**Authors:** Eyaya Misgan, Habte Belete

**Affiliations:** 1 Department of gynecology and obstetrics, College of Medicine and Health Sciences, Bahir Dar University, Bahir Dar, Ethiopia; 2 Department of psychiatry, College of Medicine and Health Sciences, Bahir Dar University, PO Box 79, Bahir Dar, Ethiopia

**Keywords:** Anxiety, Depressive, Medical illness, Primary health care, Low-income, Ethiopia

## Abstract

**Background:**

The global burden of anxiety and depressive symptoms become increasing, specifically accounts for high burden of morbidity among patients with medical conditions in low-income countries. The aim was to compare the level of anxiety and depressive symptoms in participants with general medical conditions and community residents in northwest Ethiopia.

**Methods:**

Comparatively 2625 adults in the community and 1363 patients at health center in Mecha Demographic Surveillance and Field Research Center (MDSFRC) had interviewed. Level of anxiety and depressive symptoms was assessed by Hospital Anxiety and Depression Scale (HADS) and logistic regression analysis was employed with corresponding adjusted OR (AOR) and 95% confidence interval (CI) at *p*-value less than 0.05 declaration of significant.

**Results:**

A higher prevalence of high-level anxiety and depressive symptoms, 12.6% with 95% CI; 11.0%, 14.0% and 10.1%, 95% CI; 8.0%, 12% were found among participants at health center compared to community residents, 6.8%, 95% CI; 7.0%, 8.0% and 5.2%, 95% CI; 4.0%, 6.0% at (*p* value < .0001), respectively. Social support, loss of a parent before age of 18 years, physical/verbal abuse, and having general medical conditions were significantly associated with both high-level anxiety and depressive symptoms. However, factors such as advanced age, perceived relative wealth, living alone, and having a family history of mental illness were associated with high-level of anxiety symptoms, but not with depressive symptoms.

**Conclusions:**

Proportion of high-level of anxiety and depressive symptoms were found a two-fold higher in patients with medical condition than healthy residents in the community. Patients with medical illnesses should be assess for anxiety and depressive symptoms at health center.

## Background

Neuro-psychiatric disorders, particularly high level of anxiety and depressive symptoms have received a growing attention due to their burden on quality of life [1, 2], service satisfaction [1, 3–6], medication adherence and treatment outcomes [2, 7] and impact on functionality among patients with general medical conditions at primary health care settings [8, 9]. Moreover, the proportion of high level of anxiety and depressive symptoms are very common among patients with general medical conditions in low- and high-income countries [7, 8, 10–16]. In Ethiopia, anxiety and depressive symptoms are reported up to 63.7% in patients with general medical condition and up to 10.8% in the community residents [17–19]. And demographic [20]; psychosocial [21, 22]; clinical [13, 14]; and behavioral factors were commonly reported as a risk factors of high level anxiety and depressive symptoms in previous studies [7, 13].

There is a high level of medically unexplained psychosomatic symptoms in non-psychiatric setting [23] whereas treating these somatic symptoms found to increase the recovery rate and improve functionality of patients with general medical conditions [24]. However, there is limited evidence on prevalence of high-level anxiety and depressive symptoms in patients with general medical conditions at non-psychiatric settings (health center) and in the community residents in Ethiopia, a country whose substantial health care service has been delivered at primary health care level (currently, a non-psychiatric settings). The main reason why we conduct this comparison study is to see the level and difference of anxiety and depressive symptoms among patients with general medical conditions at health center (a non-psychiatric setting) and community residents. We believe that such local epidemiological evidence is very important to develop the WHO recommended country-specific polices [25] such as to prioritize common mental health problems, to overcome the large shortage of mental health-care services throughout the country, to develop strategies that help to decrease anxiety or depression-related mortality and morbidity, and to plan integration of mental health service in primary health care system in resource limited countries like Ethiopia. Therefore, the aim of this study was to assess the prevalence and associated factors of anxiety and depressive symptoms among patients with general medical conditions at health center and community residents.

## Methods

### Participants, study design and settings

A comparative cross-sectional study has been conducted from February to March 2018 at MDSFRC in Mecha district northwest Ethiopia which is located 540 km from Addis Ababa. The study has been conducted in Merawi Health Center in Merawi area which is a semi-urban and rural. The total population in the study area is 81,000 and psychiatric service has not delivered through formal settings in the area, but available in the informal settings such as holy water and traditional medicines in the community.

All residents (resides for at least 6 months) in Merawi area were the source of population. The study population has included all adult permanent residents in the community and all patients who visit for medical attention in Merawi Health Center. All adults whose age 18 years and above, permanently reside in the community and all adult patients (age 18 years and above) who visit for medical attention in Merawi Health Center were included. Participants who couldn’t communicate well for data collectors were excluded in both groups. Participants in the comparison group live in the catchment area of the health center where the patients with general medical conditions were recruited. In the community comparison group, individuals with known any medical illness (diagnosed by health care workers) were excluded.

The sample size was determined by Epi-info version 7 software by considering 5% margin of error, 95% confidence interval, 80% power, proportion of anxiety and depressive symptoms, 10.8% for community residents [19]; 13.9% for patients with general medical conditions [26]; and 1:2 the ratio of health center to community residents. The final sample size was 4176 (1392, patients with general medical conditions and community residents, 2784).

### Data collection procedure

We administered a semi-structured interview by trained data collectors. Training manual was used to train data collectors and supervisors. Supervisors and data collectors had counterchecked the collected data for its completeness in daily base. Assuring of participants’ confidentiality and anonymity of the information were done. Systematic random sampling method used to select participants at household in the community and in the health center. For the community participants; the first household was selected by lottery method, and the rest households were selected within a constant interval. One randomly selected member of the family had taken if there were more than one eligible member in the selected one household. Data collectors had visit at convenient time to get participants for the interview and if they were not avail at their home, appointments were given two times before they were level as non-response. The interview was conducted at a separate room for all both group of participants. For health center participants, data was collected at the health center when patients come for medical attention, and had interviewed by using systematic random sampling technique.

### Variables and data collection instruments

Anxiety and depressive symptoms were measured with a score greater or equal to 8 at the Hospital Anxiety and Depression Scale (HADS) to include abnormal borderline cases in this study. HADS is a 14-item questionnaire that can be separated into two 7-item subscales for anxiety and depression. HADS is a structured tool that has good validity and reliability for screening anxiety and depressive symptoms. It has commonly used to screen for symptoms of anxiety and depression and well-validated in Ethiopia with internal consistency of 0.78 for anxiety, 0.76 for depression subscales and 0.87 for the full scale of HADS [27]. Even if the questionnaire was in English originally, it has been translated to Amharic language (local working language).

The status of current substance use has been assessed based on the participant’s report on taking of any of the following drugs: alcohol, smoking, cannabis, or khat at least once in the past 3 months [28]. Social support has been assessed by using the three items of Oslo-3 social support scale [29], which has assessed the number of close confidants, perceived level of concern from others and perceived ease of getting help from neighbors. The level of support was strong (scoring of 12–14); moderate (score of 9–11) and low (score of 3–8). History of childhood abuse was assessed by asking participants whether they had a physical or verbal abuse or not before they turn to 18 years. Family history of mental illness was assessed by asking the participants if there was any known mental illness in their parents or grandparents. Relative wealth was assessed by asking the participants what they perceived their wealth to be in relation to other people in the neighborhood [30] and were asked to level their own income by comparing with others as less than others; similar to others and better than others. Educational status was assessed based on their level of opportunity to education from not illiterate to degree and above.

### Analysis

After the data was checked for completeness and consistency, it was coded and entered in the Epi-Data 3.1 software. Data was exported to Statically Package for Social Science version 20 for analysis and *p*-value less than 0.05 was the declaration of a statistically significant. Bivariate and multiple logistic regression analyses had done to identify determinants of the outcome variables (high-level of anxiety and depressive symptoms). Bivariate logistic regression analysis was performed to examine the unadjusted relationship between the predictors and dependent variable. For independent variables which associated with outcome variables at *p*-value less than 0.05 during bivariate logistic regression analyses, multivariable logistic regression analysis was done by using backward stepwise method, and *p-*value less than 0.05 used to declare a statistically significant factor. Prevalence ratio was used to compare the outcome variable between the two group of participants. Hosmer and lemeshow test used to check the model fitness and adjusted odds ratio with a 95% confidence interval (CI) was used to indicate the strength of association.

## Results

### Response rate

A total of 3988 participants were completed the study with 95.5% response rate. From the total 4176 potential participants, 3988 had completed the interview (1363 from medical care visitors, and 2625 from community residents); however, 63 refused, 61 failed to complete the interview, and 64 were excluded due to the exclusion criteria.

### Socio-demographic information of all participants

From total participants, 1984/3988 (49.7%) of the participants were males (which composes 1271 (64.1%) from community and 713 (35.9%) from health center). The median and mean age was 34 and 35.38 (SD = 12.82) years, respectively for community sample while the median and mean age of health center samples was 32 and 35.37 (SD = 14.07) years old, respectively. See Table 1 for more details.

**Table 1:** Socio-demographic characteristics of participants

Characteristics	Overall n (%)	Sample		***P*** value	X^**2**^
		Community	Health center		
		n(%)	n(%)		
**Sex**				.02	5.4
Male	1984 (49.7)	1271 (64.1)	713 (35.9)		
Female	2004 (50.3)	1354 (67.6)	650 (32.4)		
**Living area**				<.001	367
Urban	2916 (73.1)	2174 (74.6)	742 (25.4)		
Rural	1072 (26.9)	451 (42.1)	621 (57.9)		
**Living circumstance**				=.001	10.3
Alone	243 (6.1)	183 (75.3)	60 (24.7)		
With family	3745 (93.9)	2442 (65.2)	1303 (34.8)		
**Age**				.007	12
18–24	972 (24.4)	617 (63.5)	355 (36.5)		
25–34	1170 (29.3)	795 (67.9)	375 (32.1)		
35–44	888 (22.3)	611 (68.8)	277 (31.2)		
≥ 45 years	958 (24.0)	602 (62.8)	356 (37.2)		
**Current marital status**				.04	10.1
Single	1295 (32.5)	841 (64.9)	454 (35.1)		
Married	2388 (59.9)	1599 (67.0)	789 (33.0)		
Others^a^	305 (7.6)	185 (60.7)	120 (39.3)		
**Relative wealth**				.32	2.3
Lower	1021 (25.6)	673 (65.9)	348 (34.1)		
Same	2861 (71.7)	1875 (65.9)	986 (34.5)		
Better	106 (2.7)	77 (72.6)	29 (27.4)		
**Family size**				.001	13.2
1 to 2	672 (16.9)	483 (71.9)	189 (28.1)		
3 to 4	1552 (38.9)	1006 (64.8)	546 (35.2)		
5 and more	1764 (44.2)	1136 (64.4)	628 (35.6)		

^a^(Divorced, widowed, separated)

### Clinical, substance use, lifestyle and psychological factors

From the participants who had visited for medical attention; 235/1363 (17.2%) was diagnosis for hypertension, 184/1363 (13.5%) for diabetes, 340/1363 (24.9%) for HIV/AIDS, 136/1363 (10.0%) for surgical cases, 55/1363 (4.0%) for mental illness, and 413/1363 (30.3%) for other medical conditions. Two-third of the health center sample, 1022/1363 (75.0%) had more than one-year morbidity. Current alcohol use was 739/2625 (28.2%) in community participants while 330/1363 (24.2%) in health center participants. In terms of life style, 746/2625 (28.42%) of the community sample were engaged in regular physical exercise whereas 260/1363 (19.1%) of participants in health center were engaged in regular physical exercise.

A higher proportion of childhood physical abuse (before the age of 18 years old) was reported in community samples, 1323/2625 (50.4%) than participants in health center, 637/1363 (46.7%), see Table 2.

**Table 2:** Psychosocial and behavioral characteristics of participants

Variables		Sample		***p***-value	X^**2**^ (chi-square)
		Community	Health center		
		n (%)	n (%)		
**Regular exercises**	Yes	746 (74.2)	260 (25.8)	<.001	30.288
	No	1879 (63.0)	1103 (37.0)		
**Fruits & vegetables in regular meal**	Yes	1920 (64.4)	1060 (35.6)	.001	10.169
	No	705 (69.9)	303 (30.1)		
**Social support**	Low	395 (69.8)	171 (30.2)	.001	13.063
	Average	1809 (64.1)	1014 (35.9)		
	Good	421 (70.3)	178 (29.7)		
**Depression**	Yes	132 (49.1)	137 (50.9)	<.0001	35.982
	No	2493 (67.0)	1226 (33.0)		
**Use of public health insurance**	Yes	1308 (60.5)	854 (39.5)	<.0001	59.470
	No	1317 (72.1)	509 (27.9)		
**Feeling stigmatized**	Yes	283 (72.6)	107 (27.4)	.003	8.733
	No	2342 (65.1)	1256 (34.9)		
**Physical/verbal abuse**	Yes	1323 (67.5)	637 (32.5)	.028	4.821
	No	1302 (64.2)	726 (35.8)		
**Alcohol use**	Yes	739 (69.1)	330 (30.9)	.008	7.102
	No	1886 (64.6)	1033 (35.4)		
**Smoking**	Yes	131 (72.4)	50 (27.6)	.057	3.619
	No	2494 (65.5)	1313 (34.5)		
**Khat chewing**	Yes	133 (83.6)	26 (16.4)	< .0001	23.372
	No	2491 (65.1)	1336 (34.9)		
**Cannabis use**	Yes	13 (72.2)	5 (27.8)		
	No	1611 (65.8)	1357 (34.2)		

### Prevalence of high-level of anxiety and depressive symptoms (*n* = 3988)

High-level of anxiety and depressive symptoms were found to be 8.8% (351/3988) with 95% CI; 8.0%–10.0% and 6.7% (269/3988) with 95% CI 6.0%–8.0% among total participants, respectively.

High-level of anxiety symptoms was 12.6% with 95% CI; 11.0%–14.0% among participants with general medical conditions which is significantly higher than participant from the community, 6.8% with 95% CI; 6.0%–8.0% at (*P* < .0001). High-level of depressive symptoms was 10.1% with 95% CI; 8.0%–12.0% among participants with general medical conditions which is significantly higher than participant from the community, 5.0% with 95% CI; 4.0%–6.0% at (*P <* .0001). The proportion of high-level anxiety and depressive symptoms in relation to common biopsychosocial variables is reported among participants in community and health center (Table 3).

**Table 3:** Proportion of high-level anxiety and depressive symptoms in relation to common psychosocial variables among participants

Explanatory variables	Depression Yes n(%)	Anxiety Yes n(%)	Sample	
			Community Yes n(%)	Health center Yes n(%)
**Sex**				
Male	128 (6.5)	168 (8.5)	1271 (64.1)	713 (35.9)
Female	141 (7.0)	183 (9.1)	1354 (67.6)	650 (32.4)
**Age**				
18–24	54 (5.6)	74 (7.6)	617 (23.5)	355 (26.0)
25–34	49 (4.2)	68 (5.8)	795 (30.3)	375 (32.1)
35–44	65 (7.3)	78 (8.8)	611 (23.3)	277 (31.2)
45 and more	101 (10.5)	131 (13.7)	602 (22.9)	356 (37.2)
**Living area**				
Urban	198 (6.8)	270 (9.3)	2174 (74.6)	742 (25.4)
Rural	71 (6.6)	81 (7.6)	451 (42.1)	621 (57.9)
**Living circumstance**				
Alone	17 (7.0)	29 (11.9)	183 (7.0)	60 (4.4)
With family	252 (6.7)	322 (8.6)	2442 (93)	1303 (93.9)
**Relative Wealth**				
Low	105 (10.3)	117 (11.7)	673 (65.9)	348 (34.1)
Average	152 (5.3)	216 (7.5)	1875 (65.5)	986 (34.5)
High	12 (11.3)	18 (17.0)	77 (72.6)	29 (27.4)
**Social support**				
Low	60 (10.6)	86 (15.2)	395 (69.8)	171 (30.2)
Moderate	179 (6.3)	219 (7.8)	1809 (64.1)	1014 (35.9)
Strong	30 (5.0)	46 (7.7)	421 (70.3)	178 (29.7)
**Physical/verbal abuse**				
Yes	162 (8.3)	232 (11.8)	1324 (67.6)	636 (32.4)
No	107 (5.3)	119 (5.9)	1301 (64.2)	727 (35.8)
**Parental death at childhood**				
Yes	95 (12.6)	53 (7.0)	494 (65.3)	262 (34.7)
No	174 (5.4)	298 (9.2)	2131 (65.9)	1101 (34.1)
**Family history of mental illness**				
Yes	28 (5.4)	74 (14.3)	294 (56.8)	224 (43.2)
No	241 (6.9)	277 (8.0)	2331 (67.2)	1139 (32.8)
**Alcohol use**				
Yes	89 (8.3)	121 (11.3)	739 (69.1)	330 (30.9)
No	180 (6.2)	230 (7.9)	1885 (64.6)	1032 (35.4)
**Smoking**				
Yes	10 (5.5)	13 (7.2)	131 (72.4)	50 (27.6)
No	259 (6.8)	338 (8.9)	2494 (65.5)	1313 (34.5)
**Khat chewing**				
Yes	12 (7.5)	25 (15.7)	133 (83.6)	26 (16.4)
No	257 (6.7)	326 (8.5)	2491 (65.1)	1336 (34.9)

### Determinants of high-level anxiety symptoms in community and health center

First, in a separate analysis only for participants in community, we tested factors in relation to high-level of anxiety and found that living in urban (*p* = .037, AOR .588 with 95% CI .35–.97) and higher wealth (*p* < .001, AOR .50 with 95% CI .35–.70) were a protective factor for high-level anxiety in the community sample. However, advanced age, 45 years and above (*P* < .001, AOR = 2.38 with 95% CI 1.47–3.67); poor social support (*P <* .001, AOR = 3.77 with 95% CI 2.09–6.82); physical abuse (*P <* .001, AOR = 3.38 with 95% CI 2.34–4.88), and loss of parents before age of 18 years (*P* = 003, AOR = 2.14 with 95% CI 1.30–3.54) were risk factors for high-level anxiety among community residents.

Secondly, we also tested in a separate analysis for each factors in relation to high level of anxiety among patients with medical conditions, we found that patients who had diabetes (*P* = .006, AOR = 2.33, 95% CI 1.27–4.28) and other mental illnesses (*P* < .001, AOR = 8.32, 95% CI 3.17.77); patients who be afraid dying from their current illness (*P* < .001, AOR = 2.20, 95% CI 1.50–3.20); and those who are started to take medication (*P* = .006, AOR = 2.02, 95% CI 1.23–3.33) were a risk factors of high-level anxiety. However, living in urban (*P* < .001, AOR = .47, 95% CI .32–.68) was a protective factor.

### Determinants of high-level depressive symptoms in community and health center

Similarly, in a separate analysis for community participants, we tested factors in relation to high-level of depressive symptoms and found a better wealth status (*p* < .001, AOR = .46 with 95% CI .312–.678) was a protective factor for high-level depressive symptoms in the community sample. However, age greater than 34 years (35 to 44 years (*p* = .038, AOR = 1.85 with 95% CI 1.03–3.32); 45 years and above (*P* = .001, AOR = 2.52 with 95% CI 1.45–4.37); poor social support (*P* = .024, AOR = 2.17 with 95% CI 1.11–4.26); physical abuse (*P* < .001, AOR = 2.59 with 95% CI 1.74–3.88), and loss of parents before age of 18 years (*P* < 001, AOR = 2.09 with 95% CI 1.40–3.12); participants who did not use health insurance (*p* = .001, AOR = 1.93 with 95% CI 1.31–2.85); and participants who did not do regular physical activities or exercise (*p* < .001, AOR = 4.36 with 95% CI 2.14–8.89) were risk factors for high-level depressive symptoms among community residents.

Again, we also tested each variable in relation to high level of depressive symptoms among patients with general medical conditions; current alcohol use (*P* = .001, AOR = 1.64 with 95% CI 1.12–2.40) and death of parents before the age of 18 years (*P* < .001, AOR = 2.21, 95% CI 1.5–3.27) were the only risk factors of high-level depressive symptoms.

### Significant factors for high-level anxiety in all participants (*n* = 3988)

Finally, we tested all factors in relation to high-level anxiety for both (community and health center samples) and we found that advanced age, living alone, having general medical conditions, physical abuse, and family history of mental illness were positively associated with high-level of anxiety. Moderate and strong social support, loss of a parent before age of 18 years old and lower relative wealth were negatively associated (protective) with high-level anxiety (Table 4).

**Table 4:** Associated factors of high-level of anxiety among all participants (*n =* 3988)

Explanatory variables	Anxiety		(AOR) 95% CI	***P***-value
	Yes n (%)	No n (%)		
**Age**				
18–24	74 (7.6)	898 (92.4)	1.00	
25–34	68 (5.8)	1102 (94.2)	.88 (.62, 1.26)	.490
35–44	78 (8.8)	810 (91.2)	1.42 (.99, 2.03)	.051
45 and more	131 (13.7)	827 (86.3)	2.03 (1.47, 2.80)	<.0001
**Living circumstance**				
Alone	29 (11.9)	214 (88.1)	1.57 (1.01, 2.44)	.047
With family	322 (8.6)	3423 (91.4)	1.00	
**Relative Wealth**				
Low	117 (11.7)	904 (88.5)	.45 (.26, .78)	.004
Average	216 (7.5)	2645 (92.5)	.72 (.40, 1.28)	.261
High	18 (17.0)	88 (83.0)	1.00	
**Social support**				
Low	86 (15.2)	480 (84.8)	1.00	
Moderate	219 (7.8)	2604 (92.2)	.51 (.38, .67)	<.0001
Strong	46 (7.7)	553 (92.3)	.43 (.29, .64)	<.0001
**Sample**				
Health center	172 (12.6)	1191 (87.4)	2.1 (1.67, 2.64)	<.0001
Community	179 (6.8)	2446 (93.2)	1.00	
**Physical/verbal abuse**				
Yes	232 (11.8)	1728 (88.2)	2.1 (1.63, 2.65)	<.0001
No	119 (5.9)	1909 (94.1)	1.00	
**Parental death at childhood age**				
Yes	53 (7.0)	703 (93.0)	.69 (.50, .94)	.02
No	298 (9.2)	2934 (90.8)	1.00	
**Family history of mental illness**				
Yes	74 (14.3)	444 (85.7)	1.67 (1.25, 2.23)	.001
No	277 (8.0)	3193 (92.0)	1.00	

*AOR* (Adjusted odds ratio), 1.00 (reference)

### Significant factors for high-level of depressive symptoms in all participants (*n* = 3988)

All factors in relation to high-level depressive symptoms for all participants (community and health center samples) were tested in the logistic regression and we found that having general medical conditions, loss of a parent before age of 18 years old and physical abuse were positively associated (risk) with high-level of depressive symptoms. Moderate and strong social support was negatively associated (protective) with high-level depressive symptoms (Table 5).

**Table 5:** Associated factors of high-level of depressive symptom among all participants (*n* = 3988)

Explanatory variables	Depressive symptoms		(AOR) 95% CI	***P***-value
	Yes n(%)	No n(%)		
**Age**				
18–24	54 (5.6)	918 (94.4)	1.00	
25–34	49 (4.2)	1121 (95.8)	.72 (.46, 1.14)	.157
35–44	65 (7.3)	823 (92.7)	1.29 (.79, 2.09)	.310
45 and more	101 (10.5)	857 (89.5)	1.41 (.88, 2.25)	.155
**Relative Wealth**				
Low	105 (10.3)	916 (89.7)	.543 (.28, 1.06)	.072
Average	152 (5.3)	2709 (94.7)	.84 (.42, 1.70)	.629
High	12 (11.3)	94 (88.7)	1.00	
**Social support**				
Low	60 (10.6)	506 (89.4)	1.00	
Moderate	179 (6.3)	2644 (93.7)	.631 (.45, .88)	.006
Strong	30 (5.0)	569 (95.0)	.543 (.33, .88)	.014
**Sample**				
Health center	137 (10.1)	1226 (89.9)	2.2 (1.72, 2.91)	<.0001
Community	132 (5.0)	2493 (95.0)	1.00	
**Physical/verbal abuse**				
Yes	162 (8.3)	1798 (91.7)	1.75 (1.33, 2.31)	<.0001
No	107 (5.3)	1921 (94.7)	1.00	
**Parental death at childhood age**				
Yes	95 (12.6)	661 (87.4)	2.1 (1.56, 2.77)	<.0001
No	174 (5.4)	3058 (94.6)	1.00	
**Alcohol use**				.186
Yes	89 (8.3)	980 (91.7)	1.86 (.91, 1.61)	
No	180 (6.2)	2737 (93.8)	1.00	

*AOR* (Adjusted odds ratio), 1.00 (reference)

## Discussion

The prevalence of high-level anxiety and depressive symptoms was found to be significantly higher in patients with general medical condition (12.6% and 10.1%) than community samples (6.8% and 5.0%) with prevalence ratio of 1.85 and 2.02, respectively. The adjusted logistic regression analysis also supports this result in which the odds high-level anxiety as well as depressive symptoms was two times among health center than community samples. Thus, high-level of anxiety and depressive symptoms might be occur as a result of the interaction of medical conditions with psychosocial issues that evidenced by risk of high anxiety and depressive symptoms among participants with general medical condition was found two times than community participants which is supported by previous studies [17, 18, 31]. Even if it is difficult to determine the cause-effect association in this study, participants who had general medical conditions have a higher risk of high-level anxiety and depressive symptoms than community residents.

However, the current prevalence of high-level anxiety and depressive symptoms has to be found lower compared to other similar clinical studies in central Ethiopia, 17% [8], and 18.2% in north Ethiopia [17]. The major difference for this might be due to different in measurement tools, study population such as the current study includes all patients with any medical illnesses whereas others include patients with specific chronic disorders like diabetic, HIV, hypertension, and respiratory problems which have a potential to cause high anxiety symptoms [7, 12–15]. This community prevalence also found lower than a similar community study in Ethiopia, 10.8% [19]. The main reason for this, lifetime prevalence was assessed in previous study, and participants with starting from age 15 years were included in previous study (age limit of 18 years was in our study) which some anxiety symptoms such as separation anxiety or other neurotic disorders like phobia might be predominantly had reported in previous study age group that the current study couldn’t assessed.

Advanced age was associated with high-level of anxiety (*p* < .0001), but not with high-level depressive symptoms (*p* = .155) and being in the age of 45 years and above is two times risk of high-level of anxiety symptoms than being in age 18 to 24 years old. It is predictable that anxiety symptoms can always increase with age and this finding agrees with the general understanding of this trends [20]. This is parallel to rate model study that revealed specific parts of the fear/anxiety circuit (amygdala, hippocampus and hypothalamus) in the brain undergo diverse age-related changes in response to behavioral alteration [32]. However, in contrast to this result, risk of anxiety symptoms decreases with increase of age due to possibility of decreased emotional responsiveness with age, increased emotional control and psychological immunization to stressful experiences [33]. And previous Ethiopian study had also strengthen the inversely association between age and anxiety unlike the current result [19].

Participants’ living condition has been associated with high-level of anxiety (*p* = .047), but not with high-level of depressive symptoms (*p* = .872) and living alone was found a risk factor of high-level of anxiety than living with once family. Social relationships or integration is crucial for better mental wellness over the life span [34]. Of course, as a social being, human needs belonginess as a basic need to have a good social interaction or relationships. However, loneliness can poses a significant mental health problem by increasing the risk of anxiety symptoms [35], as the current study had suggested. Loneliness may increases the chance of emerging pre-existing anxiety symptoms which is supported by similar studies [36].

Lower wealth status negatively contributes for high- level of anxiety (*p* = .004), but not for depressive symptoms (*p* = .072). The lower perceived relative wealth had found a to be protective for high-level of anxiety symptoms, up to 55% than participant with higher perceived wealth status. Which is supported by some studies [37]. Greater wealth inequality has a strong impact on the prevalence of anxiety symptoms [38]. However, in majority of the previous studies suggested that high economic and social class can prevent the occurrence of common mental disorders including high level anxiety symptoms [39] which is totally contradicted with our findings. Living with a good economic class has a substantial protection of high-level of anxiety symptoms [40] whereas low level household income had found a risk for the incidence of high-level anxiety symptoms and other common mental disorders in a prospective study [41].

High level of social support found a protective factor for both high-level of anxiety and depressive symptoms. The risk of high-level anxiety and depressive symptoms could be decrease by 49% (*p* < .0001) and 37% (*p* = .006) in moderate social support while it could be decrease by 57% (*p <* .0001) and 46% (*p* = .014) in good social support with the reference of poor social support, respectively. Good social support is very important to prevent people from high-level of anxiety and depressive symptoms, and may help people to feel confident and stability which is supported by a previous study [21]. Prospective findings justify that strong social support protected against the incidence of sever anxiety symptoms [42].

Physical abuse has found a strong risk factor for high-level of anxiety and depressive symptoms at (*p* < .0001). Participants who had experienced a history of physical abuse had found almost two times risk of high-level of anxiety and depressive symptoms than their counterparts. Physical abusive is a strong predictor of mental illness and maladaptive life events may worsen the pre-existing anxiety and depressive symptoms or may induce these symptoms as a potential risk of [22]. In some studies, the severe consequences of early physical abuse has evidenced as etiological risk of anxiety disorders [43], and high level of anxiety and depressive symptoms have been reported among adults experienced childhood physical abuse [44].

Childhood parental loss or parental lost before the age of 18 years was found as a protective factor (31%) for high-level of anxiety symptoms (*p* = .02), but found as a risk factor for depressive symptoms (*p* < .0001) than their counterparts. This is un usual finding, but also supported by some studies [45]. Parental separation or parental death has a risk of wide range adulthood psychopathology, including sever anxiety and depressive symptoms [46]. Childhood separation has a negative impact on mental wellness [47]; and different psychosocial problems such as physical abuse, poor parental care/neglection could be common in addition to lack of good parental care opportunities [22]. However, adverse childhood life events has not always associated with psychopathology [45].

Participant’s family history of mental illness was found risk of high-level of anxiety symptoms (*p* = .001) but not risk for depressive symptoms (*p* = .194) than participants without a family history of mental illness. Of course, anxiety has a strong familial association, and those children with a family history of mental illness have a greater chance of developing high-level of anxiety symptoms than their counterparts [48].

Though we had conducted a large-scale study among samples in community and primary health center, the study has a number of limitations. The first main limitation is as we did this research in two population groups, we didn’t match the participants with some important variables (sex or age) that could add value of the result. Secondly, it is difficult to determine the cause-effect association between medical illnesses and other associated factors with high-level of anxiety and depressive symptoms due to the nature of our study design. Thirdly, we did not include participants with specific medical disorders and also did not screen community participants for any medical condition, instead we only ask them whether they had a diagnosed medical condition or not.

## Conclusions

In general, high-level anxiety and depressive symptoms poses a huge burden of comorbidity at primary level health care and community residents in resource limited countries like Ethiopia. The proportions of high-level of anxiety and depressive symptoms were found significantly higher among participants in health center than in community residents. Social support, loss of a parent before age of 18 years old, physical abuse, and having general medical conditions were significantly associated with both high-level anxiety and depressive symptoms. However, factors such as advanced age, perceived relative wealth, living alone, and family history of mental illness were associated with high-level of anxiety symptoms, but not with depressive symptoms. It is recommended to assess for high-level anxiety and depressive symptoms when patients visit for medical attention at primary health cares in low-income settings. It is recommended to integrate mental health service with primary level health care system in low-income settings.

## Data Availability

The datasets used and/or analysed during the current study are available from the corresponding author on reasonable request.

## Abbreviations

AOR: Adjusted Odds Ratio
CI: Confidence Interval
HADS: Hospital Anxiety and Depression Scale
MDSFRC: Mecha Demographic Surveillance and Field Research Center
